# Choosing from Whole Cell and Acellular Pertussis Vaccines-Dilemma for the Developing Countries

**Published:** 2017-02

**Authors:** Muhammad Ali SYED

**Affiliations:** Infectious Diseases Research Group, Department of Microbiology, University of Haripur, Haripur, Pakistan

## Dear Editor-in-Chief

Pertussis or whooping cough is a highly infectious, vaccine preventable, acute respiratory tract disease. Historically, it is a disease of infants and children, but recent data reveals pertussis cases from patients of all ages and gender (1). Of particular importance are the cases of atypical, adolescent and adult pertussis, characterized by persistent cough. Both typical as well atypical pertussis have been reported from individual as well as outbreaks from highly vaccinated communities of the western world (1,2).

DTP vaccine, introduced in 1940s, has been considered as an efficacious vaccine in reducing the burden of all three diseases against that it protects. It consisted of diphtheria and tetanus toxoids and a cellular component *i.e.* inactivated cells of *Bordetella pertussis*. This whole-cell DTP vaccine (DTwP) has been replaced with acellular DTP vaccine (DTaP) consisting of individual antigens of *B. pertussis i.e.* pertussis toxin, pertactin, fimbriae, adenylate cyclase etc. The action of shift from whole cell pertussis vaccine to acellular vaccine was carried out in 1990s after reports of side effects associated with DTwP vaccines. The newly introduced acellular vaccine was less reactogenic and offer protective immunity in first 6 years of life (3).

Pertussis has been witnessed as a reemerging infectious disease in over last two decades. Many groups have attempted to find out the reasons behind the increase in reported cases of pertussis disease among vaccinated populations. Apparent reasons behind this resurgence are; waning vaccine-induced immunity, pathogen adaptation to vaccination by changing their antigenic structure, change in etiology (since pertussis-like diseases may be caused by *B. parapertussis*), and poor vaccine quality. Pertussis vaccines are not meant to protect against *B. parapertussis* infections (1,2,4,5).

There is a shift of pertussis epidemiology from infants and children to adolescent and adult population, which simply indicates waning vaccine-induced immunity among people of these age groups. Nevertheless, one cannot rule out the possibility that enhanced diagnostic facilities and surveillance system may contribute to raising in reported pertussis cases. In both cases, capability of DTaP vaccine remains questionable (1,5,6).

Rise in reported pertussis cases began soon after the introduction of acellular pertussis vaccine (DTaP) having *B. pertussis* antigens instead of whole cells. At the same time, *B. pertussis* strains undergo selection pressure and adapt to vaccination by changing their antigenic type that is different than the one used in the vaccine manufacture (5).

Pertussis cases and outbreaks are being reported from highly vaccinated populations of the western countries. Two major reasons behind vaccine failure to produce adequate level of immunity against *B. pertussis* are; waning immunity and pathogen adaptation to vaccination. New acellular vaccines are unable to offer better level of protection than the classic DTwP vaccine. They insist upon going back to whole cell vaccines due to better level of immunity conferred by them, while the others are in the favor of improving the currently used acellular vaccines (1,5,6).

As per Word Health Organization (WHO) data, majority of the pertussis cases are occurring in the developing countries of the world having highest morbidity and mortality rates. Achieving maximum vaccination coverage and maintaining the vaccine quality is a challenge, and on the other hand, real challenge is investigating vaccine efficacy. Furthermore, booster doses of DTP vaccine are not usually practices in many developing countries like Pakistan (1,6).

Many of the developing countries have already switched to acellular pertussis vaccine before getting to know the problem of waning immunity of acellular vaccines, while the others may be planning to do so. The data arriving from some incidences is really revealing. For example, highest number of pertussis cases from California outbreaks in 2010 and 2014 were infants. However, the second largest category was the older children and adolescents having average age of 10 yr (2010) and 14–16 yr (2014), which indicates waning immunity among older children and adolescents. All of them received acellular pertussis vaccines (4). Lack of surveillance data and investigation of vaccine efficacy among vaccinated people is a major constraint in choosing from both alternatives in the developing countries. The switch to acellular vaccines could have been delayed to await the response seen in the countries adapted it. Furthermore, developing countries like Pakistan should also consider booster dose and adult vaccination of those at risk and pregnant women (6).
